# Fluticasone Furoate/Vilanterol Use Trends and Characteristics in Patients With Obstructive Airway Disease: A Real-World Study of 10,374 Patients From India

**DOI:** 10.7759/cureus.34825

**Published:** 2023-02-09

**Authors:** Prahlad Prabhudesai, Bhanu P Singh, Gyanendra Agrawal, Ashok Kumar Singh, Amit Y Jadhav, Saurabh R Patil, Sagar Bhagat, Saiprasad Patil, Hanmant Barkate

**Affiliations:** 1 Chest Medicine, Lilavati Hospital and Research Center, Mumbai, IND; 2 Pulmonology, Midland Hospital, Lucknow, IND; 3 Pulmonology, Jaypee Hospital, Noida, IND; 4 Pulmonary and Critical Care Medicine, Regency Hospital, Kanpur, IND; 5 Global Medical Affairs, Glenmark Pharmaceuticals Limited, Mumbai, IND

**Keywords:** drug utilization study, ics/ultralaba, copd, asthma, fluticasone furoate/vilanterol

## Abstract

Introduction

Obstructive Airway Diseases (OADs) are the leading cause of death among chronic respiratory diseases worldwide, and novel therapies are direly needed. Fluticasone furoate/vilanterol (FF/Vi) (100/25 µg) is the first once-daily ICS/uLABA marketed in India for COPD since 2021. Considering its limited real-world experience in OAD patients in Indian clinical settings, a large drug utilization study (DUS) was planned.

Methodology

We conducted a cross-sectional, observational DUS at 1900 outpatient clinics in India from October 2021 to March 2022. Prescription data and medical history of patients who were prescribed the FF/Vi combination were collected.

Results

It was observed that FF/Vi was prescribed in an almost equal number of patients with COPD (44.2%) and asthma (42.9%). The majority of the patients (74%) were switched from previous ICS/LABA to this ICS/uLABA, while 26% of patients were treatment naïve. The average CAT score was 19.5±7.8 (43.2% GOLD Group C and 32.2% GOLD Group B) in COPD patients, while the average ACQ-5 score was 2.6±1.3 (33.1% GINA Step 3, 29.5% GINA Step 2) in asthmatic patients. Most of the patients (63.9%) had raised biomarkers (Blood eosinophil count >300 cells/μl). Prior history of exacerbation was present in 65% of patients with annual exacerbation rates of 1.2 in COPD, 1.1 in asthma, and 1.2 in asthma-COPD overlap syndrome (ACOS). Leukotriene inhibitors (42%) and LAMAs (30.8%) were common add-on medications.

Conclusion

We observed a trend towards a shift to once-daily ICS/uLABA (FF/Vi) by physicians, especially in symptomatic and exacerbating OAD patients with underlying comorbidities.

## Introduction

Obstructive Airway Diseases (OADs) are the leading cause of death among chronic respiratory diseases worldwide, with a trend towards increasing incidence [1]. The two most common OADs affecting the lung airways are chronic obstructive pulmonary disease (COPD) and asthma. The reported prevalence of COPD and asthma in India is 55.3 and 37.9 million, with each contributing to 75.6% and 20% of disability-adjusted life years (DALYs), respectively. The prevalence of asthma-COPD overlap syndrome (ACOS) is 0.3 to 5.0% in the general population [2,3].

The Global Initiative for Chronic Obstructive Lung Disease (GOLD) recommends the use of inhaled corticosteroids (ICS) in combination with bronchodilators in patients with exacerbations, a history of hospitalization, raised blood eosinophil count (BEC), concomitant asthma [4]. For asthma, ICS/LABA forms the backbone of the treatment strategy [5]. There is sufficient clinical evidence reporting the greater improvements in lung function, benefits in exacerbation rates, and quality of life, including mortality benefits in patients treated with the fixed-dose combination (FDC) of ICS and long-acting β_2_-agonist (LABA), especially those with frequent exacerbations [6-8]. 

Till 2021, only conventional ICS/LABA inhalers with twice-a-day schedules were available in India. Frequent dosing has a negative impact on adherence to prescribed inhalers, thereby increasing the risk of exacerbations. On the other hand, once daily dosing schedule has been shown to improve inhaler adherence and persistence in patients, leading to a reduction in exacerbation frequency and healthcare costs [9].

An ICS/ultra (u) LABA combination - Fluticasone furoate/Vilanterol (FF/Vi) (100/25 µg) - administered via dry powder inhaler (DPI) is the first once-daily ICS/uLABA marketed as maintenance therapy for COPD treatment in India during the study period [10,11]. However, globally it has been approved for both COPD and asthma [12,13]. 

Vilanterol (Vi), being an ultra LABA, has unique pharmacological properties as compared to other molecules of the same class. Vi shows a level of intrinsic efficacy that is significantly greater than that of salmeterol but comparable to indacaterol. It has a significantly faster onset of action and a significantly longer duration of action than salmeterol. More importantly, it has 2400 times selectivity towards β_2_ receptors reducing the potential of off-target cardiac effects [14,15]. Fluticasone furoate (FF) has high lipophilicity, high tissue permeability, low solubility, and slow inhaled drug-particle dissolution. Only around half the quantity of FF is needed to occupy the same number of glucocorticoid receptors compared to fluticasone propionate (FP). This allows for greater potency with reduced drug exposure [16,17]. Therefore, the present drug utilization study was planned to evaluate and understand the trends and characteristics of usage patterns of once-daily ICS/uLABA (FF/Vi) therapy in the Indian population.

## Materials and methods

The present study was a cross-sectional, observational drug utilization study. Before the initiation of the study, ethics committee approval was obtained from an independent ethics committee (Suraksha Ethics Committee, Asian Institute of Medical Sciences, Thane). The requirement of informed consent was waived because the data were collected retrospectively and anonymized to protect patient confidentiality. The study was conducted in compliance with the Declaration of Helsinki. The data was collected for the six months from October 2021 to March 2022 at 1900 centers across India. The collected data included demographic data, medical history, history of exacerbations, hospitalizations in the past year, and clinical features at the time of FF/Vi prescription. The severity of asthma was recorded as per steps described in GINA 2021 based on presenting symptoms, while the severity of COPD was recorded as per categories described in GOLD 2021. All prescriptions containing FF/Vi (100 μg/25 μg) with complete medical records were included, and records with missing key data were excluded from the analysis. The COPD Assessment Test (CAT) and Asthma Control Questionnaire-5 (ACQ-5) scores were used for collecting data on the symptoms of the included patients. The primary objective of the study was to evaluate the clinical use of FF/Vi in the outpatient department (OPD) of various chest clinics across India. The secondary objectives were to evaluate the use of FF/Vi in each respiratory disease, the use of reliever medications, and concomitant medications. The records were then analyzed using Microsoft Excel® (Microsoft Corporation, 2016, Redmond, USA), and descriptive statistics were generated.

## Results

**Figure 1:**
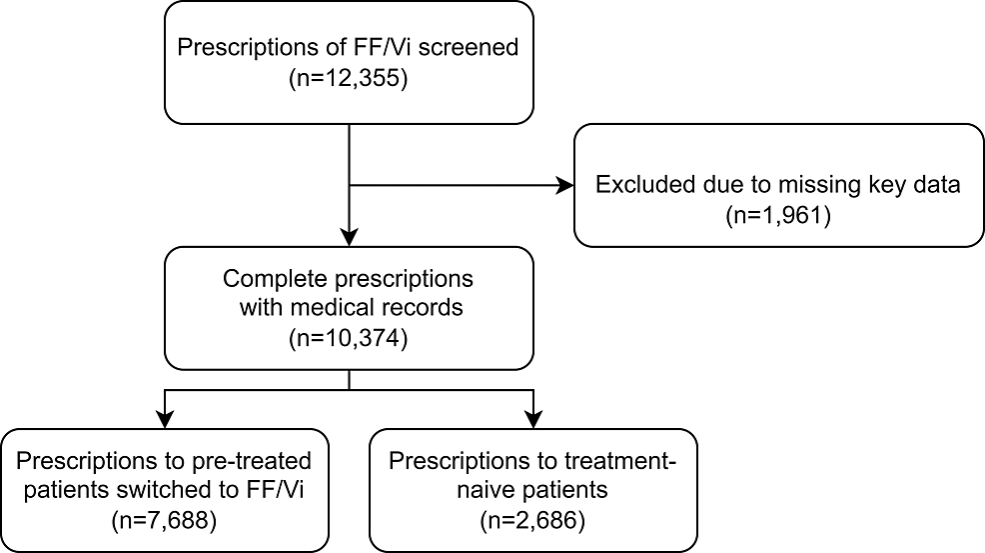
Number of medical records screened and finalized for analysis.

A total of 12,335 patients’ medical records were collected, out of which 10,374 patients' data was analyzed (Figure 1).

**Figure 2:**
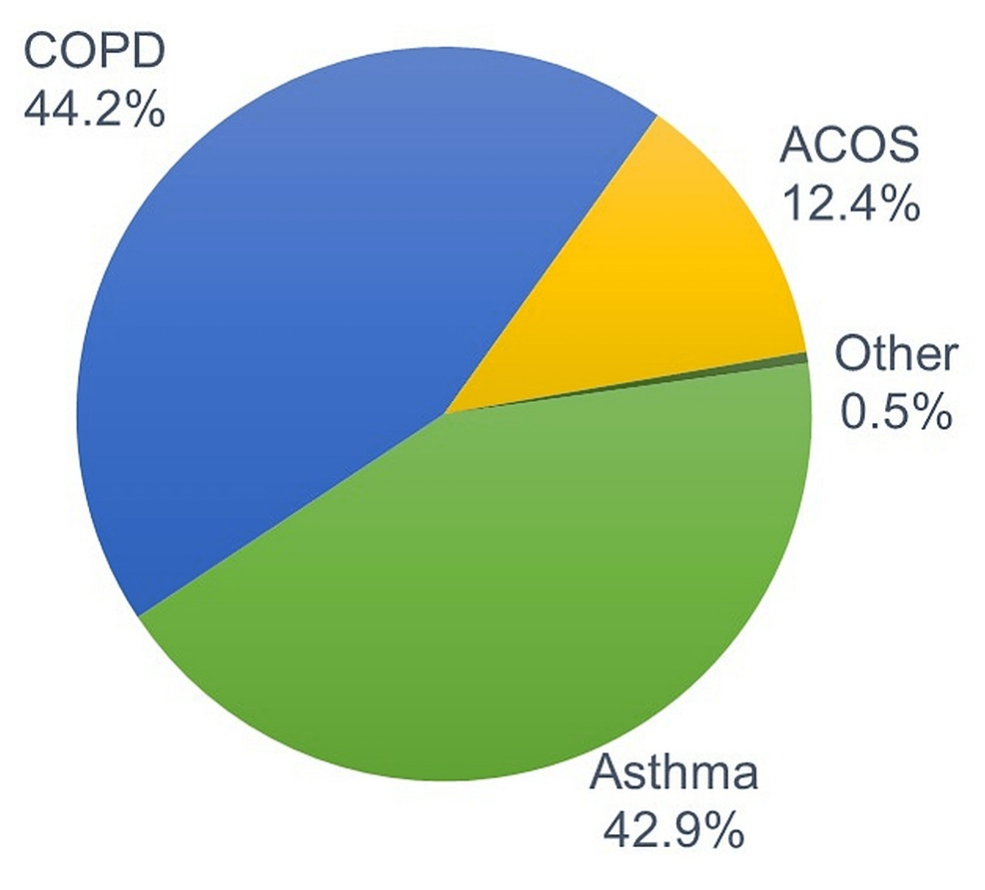
Distribution of patients to whom FF/Vi was prescribed by type of OAD.

The average age in these patients was 50.8±14.1 years, with male predominance (68.1%). The most common indications for which FF/Vi was prescribed were COPD (44.2%) and asthma (42.9%), with the rest being ACOS (12.4%) (Figure 2).

The average duration of disease in COPD and ACOS patients was 8.95 ± 7.11 years and 8.34 ± 7.83 years, respectively, while in asthma, it was 5.74 ± 5.90 years. The most common comorbidities in COPD patients were diabetes mellitus (40%) and cardiovascular diseases (26.7%), whereas the asthma patients had GERD (19.7%) and obesity (18.7%) as associated common comorbidities. Patients with ACOS show higher incidences of diabetes mellitus (31.4%), cardiovascular diseases (28.8%), and obesity (28.8%) compared to those without ACOS (Table 1).

**Table 1:** Details of the group-wise distribution of exacerbations

Number of exacerbations in past year (N=6719)		
ACOS (N=909, 13.5%)	<2	296 (32.6%)
	2-5	562 (61.8%)
	>5	51 (5.6%)
Asthma (N=2434, 36.2%)	<2	1206 (49.5%)
	2-5	1135 (46.6%)
	>5	93 (3.8%)
COPD (N=3376, 50.3%)	<2	1212 (35.9%)
	2-5	1980 (58.6%)
	>5	184 (5.5%)

A majority of the COPD patients belonged to Group C (43.2%) and Group B (32.2%), with an average CAT score of 19.5±7.8, indicating highly symptomatic status. Most of the asthma patients belonged to Step 3 (33.1%) and Step 2 (29.5%) with an average ACQ-5 score of 2.6±1.3, indicating uncontrolled status. Overall, most of the patients had a high BEC (>300 per µl, 63.9%). Of these, patients with asthma (66 %) had the highest proportion of high BEC, followed by ACOS (60.5%) and COPD (57.8%). The majority of the patients were initially on conventional ICS/LABA inhalers, with the proportion being highest in asthmatics (90.7%), followed by ACOS (69.6%) and COPD (61.6%). Some patients were on ICS/LABA/LAMA triple therapy in COPD (26.1%) and ACOS (22.6%); however, very few (6.8%) asthmatics were on it. The analysis of the overall presence of exacerbations and their frequency in the past year revealed that two-thirds of the patients (about 65%) had a history of exacerbation, with a third of them (35.6%) having two to five episodes in the past year and few (4.9%) had more than five episodes in last one year. A disease-wise distribution of exacerbations showed that several patients with asthma (49.5%) had a history of fewer than two exacerbations, while in ACOS and COPD, the numbers of low exacerbations were lower, 32.6% and 35.9%, respectively. (Table 1). The group-wise pattern of the number of hospitalizations due to exacerbations was similar in all three groups (Table 2).

**Table 2:** Details of the group-wise distribution of hospitalization due to exacerbations

Number of exacerbations in past year (N=3391)		
ACOS (N=492, 14.5%)	<2	324 (65.9%)
	2-3	156 (31.7%)
	4-5	7 (1.4%)
	>5	5 (1.0%)
Asthma (N=920, 27.1%)	<2	755 (82.1%)
	2-3	157 (17.1%)
	4-5	7 (0.8%)
	>5	1 (0.1%)
COPD (N=1979, 58.4%)	<2	1357 (68.6%)
	2-3	589 (29.8%)
	4-5	23 (1.2%)
	>5	10 (0.5%)

**Figure 3:**
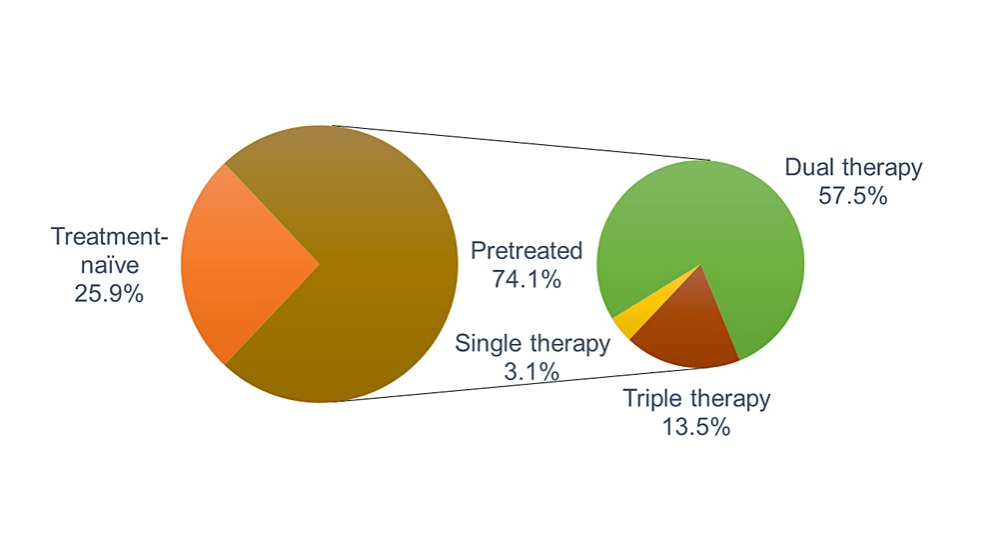
Pattern of switching patients to FF/Vi. Most of the patients were switched to FF/Vi from dual therapy, with some from triple therapy

One-fifth of patients (22.1%) had a history of pneumonia. In the present study, three-fourths (74.1%) of the patients were on prior medications and were switched to FF/Vi treatment, while 25.9% of patients were treatment naïve. The switched-over patients were on either mono, dual, or triple therapy before their inclusion in the study. The prevailing switch was observed from dual and triple therapy, with most of them (57.5%) from dual therapy (Figure 3).

**Figure 4:**
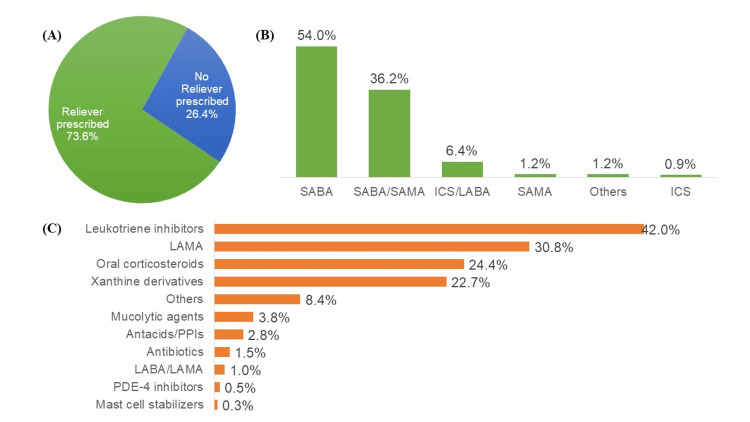
A) Proportion of patients prescribed reliever medication. B) Pattern of prescribed reliever medications with FF/Vi. C) Pattern of prescription of concomitant drugs with FF/Vi.

For all three groups, most of the patients switched to FF/Vi from prior conventional twice-a-day ICS/LABA therapy. The most common ICS/LABA combinations for switched prescriptions were Budesonide/Formoterol (60.46%) in the dual regimen and Budesonide/Formoterol/Tiotropium (7.3%) in the triple therapy. Most of the patients used DPI (66.6%), followed by MDI (32.9%). Reliever medication was prescribed in 73.6% (Figure 4a) of the total cases. SABAs (54.0%) were the most frequently prescribed class of medication as a reliever (Figure 4b), and Levosalbutamol (31.13%) was the most common reliever medication prescribed. The commonly prescribed concomitant medications, along with their most common molecules, were Leukotriene inhibitors (42.0%): Montelukast (51.7%); LAMA (30.8%): Glycopyrronium (66.8%); Oral corticosteroids (24.4%): Deflazacort (21.0%) and Xanthine derivatives (22.7%): Acebrophylline (63.6%) (Figure 4c).

## Discussion

In the current study, it was reflected that FF/Vi (100 µg/25 µg) DPI was prescribed for COPD, asthma, as well as ACOS in clinical practice. In various landmark clinical studies once-daily, FF/Vi had shown significant efficacy with optimized symptom control, reduced exacerbation risk, reduced all-cause mortality, and improved adherence in OADs, including ACOS, as compared to conventional dual therapies [10,18-25]. The British Thoracic Society (BTS) Guidelines have included Fluticasone furoate and LABA therapy for the management of asthma as it reduces exacerbations and leads to improvement in lung function. 

Gross et al. (2015) assessed the ethnic sensitivity of FF/Vi in the East Asian Asthma population and observed good efficacy and a good safety profile for FF/Vi (100/25 μg and 200/25 μg) OD in patients. OAD often coexists with other chronic comorbidities, which have a major impact on health status and prognosis. In India, 60% of COPD patients had CVD comorbidities [26]. In the current study, diabetes mellitus, obesity, cardiovascular disease, and GERD were found to be the prevalent comorbidities. Covelli et al. (2015) reported the efficacy of FF/Vi in improving lung function in COPD patients with CVD risk factors [21,27]. In a landmark trial for FF/Vi in COPD (SUMMIT trial), patients with moderate COPD and a history of CVD or at increased risk for CVD had significantly lowered all cause-mortality risk (p=0.0158, HR=0.76) and a 7.4% decrease in cardiac events [20]. In the Lung Deflation Study by Stone et al. (2016), there was evidence that indicates that FF/Vi use resulted in an improvement in the cardiac index by +0.203 L/min/m2 (p=0.004) with significant improvements in ejection fraction (p=0.0001) [27]. 

A majority of the patients (65%) in the present study had a history of exacerbation in the previous year. In practice, SABA overuse is common. SABINA studies have generated evidence that such overuse is associated with adverse outcomes, including an increase in acute exacerbations of the disease [28,29]. Vestbo et al. (2016) determined that a once-daily treatment regimen of FF/Vi lowered the rate of exacerbations in the usual care group (BUD/FOR or FP/SAL) in patients with COPD and a history of exacerbations [18]. Not only this but in FP/SAL subset analysis, FF/Vi has shown a 20% significant reduction in severe exacerbations, significant benefit in asthma control, quality of life, and reduced reliever medication use than continuing FP/SAL [21]. Bakerly ND et al. (2021) also concluded that FF/Vi may represent a less costly alternative in direct COPD-related total medical costs to current therapies in such patients [30]. The CAPTAIN study, another phase III landmark trial, demonstrated clinically important improvements from baseline in Asthma Control Questionnaire-7 (ACQ-7), St. George's Respiratory Questionnaire (SGRQ), and Evaluating Respiratory Symptoms in Asthma (E-RS: Asthma) total scores in all pooled groups (FF/Vi, 100/25 and 200/25) [31]. However, the IMPACT trial, which was done in patients with symptomatic COPD (COPD Assessment Test [CAT] score ≥10) and ≥1 moderate/severe exacerbation in the prior year, indicated that irrespective of baseline CAT score, FF/Vi reduced the annual moderate/severe exacerbation rates and had overall favorable profile over dual therapy [32]. Various studies have shown that ACOS patients have significantly poor outcomes as compared with patients with asthma or COPD alone [2,33]. Around 61.82% of patients with a history of exacerbation belonged to the ACOS group in the current study. Ishiura et al. (2015) provided substantial evidence for the utilization of FF/Vi in the regular treatment of ACOS [23]. Growing evidence suggests that BEC could help predict the response to ICS, assisting clinicians in treatment selection. Pascoe et al. (2015) found that as compared with vilanterol alone, reductions in exacerbations with FF/Vi were 42% for those with BEC of ≥6% [34]. In the present study, despite the history of pneumonia in the past year, this combination was preferred as it is likely to reduce future exacerbations. In the CAPTAIN study, in patients with raised type-2 inflammatory biomarkers (BEC >300 cells and FeNO >50 ppb), high-dose FF/Vi has shown a significant reduction in exacerbations as well as all cause-mortality compared to medium dose and add-on LAMA [31,35]. This is suggestive of the preference for FF/Vi in patients with the raised BEC by prescribers. 

It was observed that most of the patients switched to FF/Vi from prior conventional ICS/LABA therapy in all three groups COPD, asthma, and ACOS. SABAs were the most commonly used reliever medication class when on regular treatment with FF/Vi, which is in line with recommendations of GINA track 2. Shimizu et al. (2020) evaluated the efficacy of switching therapy from fluticasone propionate/salmeterol (FP/SAL) or budesonide/formoterol (BUD/FOR) to FF/Vi at the equivalent corticosteroid dose and found that FEV1 was improved at four weeks after switching, and the improvement was maintained till 12 weeks [36]. Averell et al. (2018) found that initiating once-daily FF/Vi had higher adherence and were less likely to discontinue therapy in asthma patients as compared with patients initiating either twice-daily BUD/FOR or FP/SAL [37]. A study by Woodcock et al., 2017 has shown that FF/Vi has a significant benefit on asthma symptom control as well as responder rate compared to conventional ICA/LABA; this would be one of the reasons for switching from BUD/FOR and FP/SAL to once-daily FF/Vi in the current study [18,19]. This study showed that LAMA, especially once-daily glycopyrronium, was the most commonly prescribed add-on medication to once-daily FF/Vi with dosing flexibility in the morning and evening. Contrary to the conventional twice-a-day ICS/LABA, which increases the dosing frequency and impacts adherence and compliance in these populations. 

There are a few limitations to the current study, including retrospective research, and data collection was based on medical records. This study was cross-sectional and did not have a longitudinal follow-up to evaluate treatment responses over some time.

## Conclusions

FF/Vi combination was prescribed across the spectrum of OADs: COPD, asthma, and ACOS. There is an increasing trend towards switching patients to FF/Vi therapy in uncontrolled, exacerbating, and symptomatic OAD patients with various comorbidities, which might be attributable to its previously reported evidence in reducing exacerbations, symptom control, and cardiovascular safety.
